# Supplementary material for: RCE-IFE: recursive cluster elimination with intra-cluster feature elimination
Source: PeerJ Comput Sci. 2025 Feb 7;11:e2528. doi: 10.7717/peerj-cs.2528 (PMC11888879; doi:10.7717/peerj-cs.2528)
Supplement: Supplemental Information 2 [file peerj-cs-11-2528-s002.docx]

|  | Specificity | | | Precision | | |
| --- | --- | --- | --- | --- | --- | --- |
|  | SVM-RCE | RCE-IFE-SVM | RCE-IFE | SVM-RCE | RCE-IFE-SVM | RCE-IFE |
| GDS1962 | 0.93 ± 0.21 | 0.92 ± 0.18 | 0.92 ± 0.20 | 0.97 ± 0.07 | 0.97 ± 0.07 | 0.97 ± 0.07 |
| GDS2519 | 0.50 ± 0.21 | 0.54 ± 0.22 | 0.51 ± 0.20 | 0.45 ± 0.18 | 0.52 ± 0.18 | 0.46 ± 0.17 |
| GDS2547 | 0.71 ± 0.16 | 0.72 ± 0.16 | 0.71 ± 0.17 | 0.73 ± 0.12 | 0.75 ± 0.12 | 0.73 ± 0.13 |
| GDS2609 | 0.93 ± 0.26 | 0.97 ± 0.17 | 0.94 ± 0.24 | 0.97 ± 0.09 | 0.99 ± 0.07 | 0.98 ± 0.09 |
| GDS3268 | 0.73 ± 0.17 | 0.72 ± 0.19 | 0.64 ± 0.19 | 0.76 ± 0.13 | 0.76 ± 0.14 | 0.71 ± 0.12 |
| GDS3646 | 0.38 ± 0.34 | 0.48 ± 0.36 | 0.37 ± 0.36 | 0.78 ± 0.13 | 0.81 ± 0.13 | 0.77 ± 0.12 |
| GDS3794 | 0.90 ± 0.23 | 0.90 ± 0.23 | 0.88 ± 0.23 | 0.93 ± 0.15 | 0.93 ± 0.15 | 0.91 ± 0.17 |
| GDS3837 | 0.95 ± 0.09 | 0.94 ± 0.09 | 0.94 ± 0.09 | 0.96 ± 0.07 | 0.95 ± 0.08 | 0.95 ± 0.08 |
| GDS3874 | 0.52 ± 0.28 | 0.52 ± 0.32 | 0.58 ± 0.29 | 0.75 ± 0.13 | 0.75 ± 0.15 | 0.80 ± 0.13 |
| GDS3875 | 0.67 ± 0.28 | 0.63 ± 0.27 | 0.56 ± 0.32 | 0.83 ± 0.14 | 0.81 ± 0.13 | 0.79 ± 0.14 |
| GDS3929 | 0.07 ± 0.21 | 0.04 ± 0.15 | 0.18 ± 0.27 | 0.68 ± 0.07 | 0.66 ± 0.06 | 0.65 ± 0.13 |
| GDS4228 | 0.07 ± 0.17 | 0.04 ± 0.13 | 0.45 ± 0.36 | 0.67 ± 0.05 | 0.66 ± 0.04 | 0.78 ± 0.13 |
| GDS4824 | 0.88 ± 0.33 | 0.93 ± 0.26 | 0.90 ± 0.30 | 0.91 ± 0.23 | 0.96 ± 0.14 | 0.91 ± 0.25 |
| GDS5037 | 0.51 ± 0.38 | 0.51 ± 0.34 | 0.47 ± 0.36 | 0.82 ± 0.14 | 0.81 ± 0.12 | 0.82 ± 0.11 |
| GDS5093 | 0.68 ± 0.47 | 0.73 ± 0.45 | 0.69 ± 0.46 | 0.88 ± 0.18 | 0.90 ± 0.17 | 0.88 ± 0.18 |
| GDS5499 | 0.84 ± 0.18 | 0.83 ± 0.20 | 0.77 ± 0.19 | 0.94 ± 0.07 | 0.93 ± 0.08 | 0.90 ± 0.07 |
| GSE157103 | 0.87 ± 0.19 | 0.87 ± 0.21 | 0.80 ± 0.21 | 0.93 ± 0.10 | 0.93 ± 0.10 | 0.89 ± 0.11 |
| TCGA-BLCA.methylation450 | 0.84 ± 0.26 | 0.90 ± 0.21 | 0.89 ± 0.22 | 0.94 ± 0.10 | 0.96 ± 0.08 | 0.95 ± 0.10 |
| TCGA-BLCA.mirna | 0.94 ± 0.18 | 0.89 ± 0.22 | 0.89 ± 0.22 | 0.97 ± 0.07 | 0.95 ± 0.09 | 0.95 ± 0.09 |
| TCGA-BRCA.methylation450 | 0.97 ± 0.06 | 0.97 ± 0.06 | 0.98 ± 0.05 | 0.95 ± 0.09 | 0.96 ± 0.09 | 0.98 ± 0.07 |
| Average | 0.69 ± 0.23 | 0.70 ± 0.22 | 0.70 ± 0.25 | 0.84 ± 0.12 | 0.85 ± 0.11 | 0.84 ± 0.12 |
