# Supplementary material for: RCE-IFE: recursive cluster elimination with intra-cluster feature elimination
Source: PeerJ Comput Sci. 2025 Feb 7;11:e2528. doi: 10.7717/peerj-cs.2528 (PMC11888879; doi:10.7717/peerj-cs.2528)
Supplement: Supplemental Information 4 [file peerj-cs-11-2528-s004.docx]

| Metric | Method | CRC_enzyme | CRC_pathway | CRC_species | CRC_species_II | IBD | IBDMDB | T2D | Average |
| --- | --- | --- | --- | --- | --- | --- | --- | --- | --- |
| Accuracy | RCE-IFE | 0.69 ± 0.04 | 0.64 ± 0.04 | 0.74 ± 0.04 | 0.74 ± 0.14 | 0.79 ± 0.07 | 0.90 ± 0.03 | 0.62 ± 0.09 | 0.73 ± 0.06 |
|  | SVM-RCE | 0.65 ± 0.04 | 0.64 ± 0.04 | 0.62 ± 0.05 | 0.51 ± 0.16 | 0.75 ± 0.07 | 0.74 ± 0.03 | 0.56 ± 0.09 | 0.64 ± 0.07 |
| Sensitivity | RCE-IFE | 0.66 ± 0.06 | 0.62 ± 0.06 | 0.66 ± 0.07 | 0.82 ± 0.18 | 0.82 ± 0.10 | 0.97 ± 0.02 | 0.63 ± 0.14 | 0.74 ± 0.09 |
|  | SVM-RCE | 0.56 ± 0.06 | 0.60 ± 0.07 | 0.45 ± 0.12 | 0.51 ± 0.32 | 0.69 ± 0.11 | 0.94 ± 0.03 | 0.64 ± 0.22 | 0.63 ± 0.13 |
| Specificity | RCE-IFE | 0.71 ± 0.05 | 0.66 ± 0.06 | 0.82 ± 0.05 | 0.67 ± 0.23 | 0.75 ± 0.12 | 0.77 ± 0.07 | 0.62 ± 0.13 | 0.71 ± 0.10 |
|  | SVM-RCE | 0.73 ± 0.06 | 0.68 ± 0.07 | 0.79 ± 0.10 | 0.52 ± 0.33 | 0.81 ± 0.10 | 0.35 ± 0.10 | 0.47 ± 0.20 | 0.62 ± 0.14 |
| Precision | RCE-IFE | 0.69 ± 0.05 | 0.64 ± 0.05 | 0.78 ± 0.05 | 0.74 ± 0.16 | 0.78 ± 0.09 | 0.90 ± 0.03 | 0.62 ± 0.09 | 0.74 ± 0.07 |
|  | SVM-RCE | 0.68 ± 0.05 | 0.65 ± 0.05 | 0.69 ± 0.10 | 0.51 ± 0.24 | 0.79 ± 0.08 | 0.75 ± 0.03 | 0.54 ± 0.11 | 0.66 ± 0.09 |
| F-measure | RCE-IFE | 0.68 ± 0.05 | 0.63 ± 0.05 | 0.71 ± 0.05 | 0.76 ± 0.13 | 0.79 ± 0.07 | 0.93 ± 0.02 | 0.62 ± 0.10 | 0.73 ± 0.07 |
|  | SVM-RCE | 0.61 ± 0.05 | 0.62 ± 0.05 | 0.53 ± 0.11 | 0.53 ± 0.18 | 0.73 ± 0.08 | 0.83 ± 0.02 | 0.58 ± 0.14 | 0.63 ± 0.09 |
| Cohen’s kappa | RCE-IFE | 0.37 ± 0.08 | 0.28 ± 0.09 | 0.48 ± 0.07 | 0.49 ± 0.27 | 0.57 ± 0.15 | 0.77 ± 0.07 | 0.25 ± 0.18 | 0.46 ± 0.13 |
|  | SVM-RCE | 0.30 ± 0.08 | 0.27 ± 0.08 | 0.24 ± 0.11 | 0.03 ± 0.31 | 0.50 ± 0.14 | 0.33 ± 0.10 | 0.11 ± 0.19 | 0.25 ± 0.14 |
